# Data mining and safety analysis of avatrombopag: a retrospective pharmacovigilance study based on the US food and drug administration’s adverse event reporting system

**DOI:** 10.1038/s41598-024-62129-5

**Published:** 2024-05-17

**Authors:** Hong Zhu, Meng Wu

**Affiliations:** 1 Department of Pharmacy, The Affiliated Huaian No.1 People’s Hospital of Nanjing Medical University, Huaian, 223300 Jiangsu China; 2 Department of Oral and Maxillofacial Surgery, The Affiliated Huaian No.1 People’s Hospital of Nanjing Medical University, 1 Huanghe West Road, Huaian, 223300 Jiangsu China

**Keywords:** Avatrombopag, Thrombopoietin receptor agonist, FAERS, Adverse event, Haematological diseases, Drug safety, Drug discovery

## Abstract

With its increasing use in the treatment of thrombocytopenia, avatrombopag’s associated adverse events (AEs) pose a major challenge to its clinical application. This study aims to comprehensively study AEs associated with avatrombopag by using real-world evidence. We curated AE reports for avatrombopag from the first quarter of 2018 to the fourth quarter of 2023 in the US Food and Drug Administration’s Adverse Event Reporting System (FAERS) database. AEs were coded using the Medical Dictionary for Regulatory Activities of Preferred Terms and System Organ Classes. The reporting odds ratio, proportional reporting ratio, Bayesian confidence propagation neural network, and multi-item Gamma-Poisson Shrinker were used to investigate the relationship between avatrombopag and AE reports. Among 9,060,312 reported cases in the FAERS database, 1211 reports listed avatrombopag as “primary suspected” drug. Disproportionality analysis identified 44 preferred terms across 17 organ systems met the criteria for at least one of the four algorithms. The most commonly reported AEs were platelet count decreased (20.2%), headache (16.7%), platelet count increased (11.9%), platelet count abnormal (6.3%), contusion (2.7%), pulmonary embolism (2.3%), and deep vein thrombosis (2.1%). Unexpected AEs such as seasonal allergy, rhinorrhea, antiphospholipid syndrome, ear discomfort, and photopsia were also observed. Excluding the other serious outcomes, hospitalization (34.6%) was the most frequently reported serious outcome, followed by death (15.4%). Most reported AEs occurred within the first 2 days of initiating avatrombopag therapy, and the median onset time was 60 days. We identified new and unexpected AEs with clinical use of avatrombopag, and our results may provide valuable information for clinical monitoring and identifying risks associated with avatrombopag.

## Introduction

Thrombocytopenia is a common hematological condition that can result from various etiologies such as chemotherapy-induced thrombocytopenia, myelodysplastic syndrome, chronic liver disease, and immune thrombocytopenia (ITP)^1^. Bleeding is the most common clinical manifestation and can range from mild and common (e.g., petechiae and ecchymoses) to severe (e.g., visceral or intracranial hemorrhage)^2^. Symptomatic platelet transfusion is the most effective treatment for acute severe thrombocytopenia–induced bleeding; however, a limited platelet supply, transfusion reactions caused by repeated platelet transfusions, and ineffective platelet transfusion limit the clinical application of platelets^3^. In recent years, the development of platelet-stimulating agents such as thrombopoietin (TPO) and thrombopoietin receptor agonists (TPO-RAs) has received increasing attention.

TPO-RAs are second-generation thrombopoietic drugs that bind to the TPO receptor, thereby altering its conformation and activating the JAK2/STAT5 pathway and increasing the proliferation of megakaryocytes and myeloid progenitor cells and, subsequently, platelet production^4^. Newly developed second-generation TPO-RAs can significantly increase platelet counts and reduce the incidence of total or serious bleeding events, thus lowering the dose of platelet transfusion and improving patients’ quality of life^5^. TPO-RAs mainly include TPO peptide mimetics (e.g., romiplostim), and TPO nonpeptide mimetics^6^. The primary TPO nonpeptide mimetics currently used clinically are avatrombopag and eltrombopag, which, since being approved for use, are now used in more than 100 countries^4^. The pharmacodynamics and pharmacokinetics of TPO-RAs differ owing to their molecular structures. Avatrombopag, which was approved for marketing in the United States in 2018, has more advantages over romiplostim and eltrombopag^7–9^, such as no black box warning for hepatotoxicity^10^ and non-requirement of dietary restrictions^11^. Alternatively, avatrombopag is more convenient to take orally than romiplostim, which must be administered subcutaneously^12^. Hence, avatrombopag is gradually replacing other ITP treatment agents, having resulted in durable and stable platelet responses^10^.

Despite its outstanding outcomes in clinical application, avatrombopag’s side effects should be taken seriously. In a phase 3 randomized clinical study of avatrombopag, the overall incidence of adverse events (AEs) in the avatrombopag-treated group was 96.9%, which was markedly higher than that (58.8%) reported in the placebo group; nevertheless, there was no significant variation in incidence rates after adjusting for exposure in a clinical setting^13^. Clinical studies on AEs related to avatrombopag have mostly been limited by relatively small sample sizes, selection criteria, and limited follow-up duration.

The United States Food and Drug Administration (FDA) Adverse Event Reporting System (FAERS), one of the world's largest public pharmacovigilance databases, aids the FDA in monitoring drug and therapeutic product safety after they are available in the market^14^. Manufacturers must submit all AE reports to the FDA, whereas health care professionals and consumers worldwide may do so voluntarily. Given the extensive utilization of avatrombopag in real-world settings and the limited assessments of its associated AEs, we performed a pharmacovigilance study to assess the safety characteristics of avatrombopag and inform clinicians on rational drug use.

## Materials and methods

### Data sources and procedures

**Figure 1:**
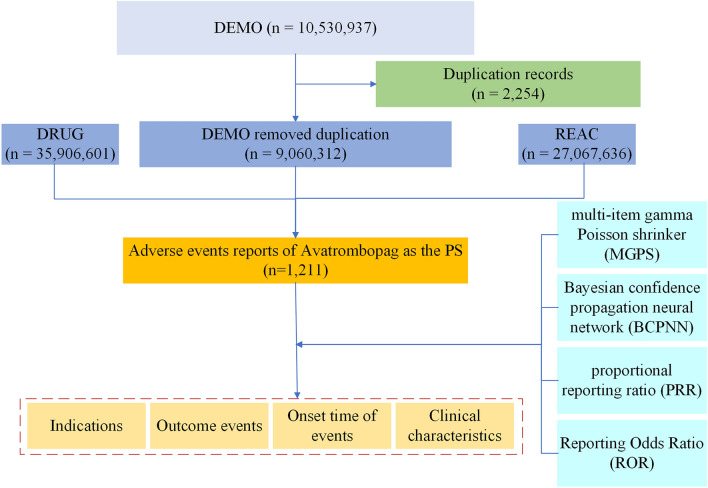
A flowchart of the participant selection process.

We performed a retrospective pharmacovigilance study using data from the FAERS quarterly data from the first quarter of 2018 (January 2018) to the fourth quarter of 2023 (December 2023). The participant selection process is shown in Fig. 1. We use a combination of generic and proprietary drug names to identify cases. AVATROMBOPAG MALEATE, AVATROMBOPAG, and DOPTELET were used as search names for avatrombopag to search for reported adverse events. Since the database is voluntary reporting, there will be duplicate reports. In our study, the raw data performed a de-duplication process (n = 2254), after which we created a dataset caused by avatrombopag as the primary suspected (PS) drug report. This study complied with the tenets of the Declaration of Helsinki, and all methods were performed in accordance with relevant guidelines. AEs were coded using the Medical Dictionary for Regulatory Activities (MedDRA) at the Preferred Term (PT; version 25.0)^15^ level. Only drugs reported as “a primary suspect product” were included in the analysisb. AEs and AE categories were defined as PTs and “System Organ Classes (SOCs),” respectively. Dates of AE occurrence and initiation of avatrombopag treatment were used to determine the onset time.

### Statistical analysis and signals detection

We used R software (version 4.1.0; R Foundation for Statistical Computing) to statistically compute disproportionality signals. Data mining was performed using the reporting odds ratio, proportional reporting ratio, Bayesian confidence propagation neural network, and multi-item Gamma-Poisson shrinker in a disproportionality analysis^16^. Student’s t-test was used to determine the P-value for comparison of number of people between groups at different times of AE onset. Supplementary Table S1 contains the formulae and requirements for each of the four algorithms. AE signals were defined as signals that could be detected by all 4 methods and exhibited a statistical correlation between the target drug and the target AE. AEs have been disproportionately reported in association with avatrombopag.

## Results

### Overview

From the first quarter of 2018 to the fourth quarter of 2023, this study obtained a total of 10,530,937 adverse event reports from the FAERS database. After removing duplicates of 2254, of the 9,060,312 cases reported, 1211 reports listed avatrombopag as “primary suspected” drug. An overview of AEs reported in association with avatrombopag is provided in Table 1. Women (54.3%) accounted for a larger proportion of AEs than men. Patients aged ≥ 65 years accounted for a larger proportion (22.2%) of participants. The largest number of AEs was reported in the United States (88.2%), followed by Spain (2.1%), Italy (1.8%), China (1.7%), and Australia (0.8%). Serious outcomes included hospitalization, death, life-threatening conditions, disability, and other serious outcomes. Excluding the other serious outcomes, hospitalization (34.6%) was the most frequently reported serious outcome, followed by death (15.4%). Consumers, physicians, and health professionals reported the most AEs (42.3%, 26.0%, and 24.6%, respectively). AEs were reported in 2018 (n = 55, 4.5%), 2019 (n = 110, 9.1%), 2020 (n = 262, 21.6%), 2021 (n = 257, 21.2%), 2022 (n = 245, 20.2%), and 2023 (n = 282, 23.3%).

**Table 1:** Clinical characteristics of reports with avatrombopag from the FAERS database (January 2018 to December 2023).

Characteristics	Reports, n (%) N = 1211
Sex	
Female	658 (54.3%)
Male	463 (38.2%)
Unknown	90 (7.4%)
Weight (kg)	
< 50	6 (0.5%)
50 ~ 100	132 (10.9%)
> 100	47 (3.9%)
Unknown	1026 (84.7%)
Age (years)	
≤ 17	8 (0.7%)
18 ~ 64	258 (21.3%)
≥ 65	268 (22.1%)
Unknown	677 (55.9%)
Occupation of reporter	
Consumer	512 (42.3%)
Physician	315 (26.0%)
Health profession	298 (24.6%)
Other health-professional	58 (4.8%)
Pharmacist	25 (2.1%)
Unknown	3 (0.2%)
Serious outcome	
Death	76 (15.4%)
Hospitalization	170 (34.6%)
Life-threatening	7 (1.4%)
Disability	2 (0.4%)
Other serious outcome	237 (48.2%)
Report countries (Top five)	
America	1068 (88.2%)
Spain	26 (2.1%)
Italy	22 (1.8%)
China	21 (1.7%)
Australia	10 (0.8%)
Reporting year	
2023	282 (23.3%)
2022	245 (20.2%)
2021	257 (21.2%)
2020	262 (21.6%)
2019	110 (9.1%)
2018	55 (4.5%)

### Signal detection

In Table 2, potential signals for avatrombopag are described in accordance with the SOC. Statistics show that 26 organ systems were affected by reported AEs. No potential signals satisfied our signal criteria when AE reports were classified at the SOC level. Significant potential signals for the nervous system disorders, general disorders and administration site conditions, vascular disorders, investigations, and hepatobiliary disorders SOCs were identified for at least one of the four disproportionality indices.

**Table 2:** Reporting potential signals for avatrombopag at the System Organ Class (SOC) level in FAERS database.

System organ class (SOC)	Avatrombopag cases reporting SOC	ROR (95% two-sided CI)	PRR (χ^2^)	IC (IC 025)	EBGM (EBGM 05)
Nervous system disorders (SOC:10,029,205)	318	1.46 (1.3–1.63)*	1.41 (40.51)	0.49 (− 1.17)	1.41 (1.25)
General disorders and administration site conditions (SOC: 10,018,065)	626	1.23 (1.12–1.34)*	1.18 (20.92)	0.24 (− 1.43)	1.18 (1.08)
Gastrointestinal disorders (SOC: 10,017,947)	268	1.12 (0.99–1.27)	1.11 (2.98)	0.15 (− 1.52)	1.11 (0.98)
Skin and subcutaneous tissue disorders (SOC: 10,040,785)	110	0.6 (0.5–0.73)	0.62 (28.1)	− 0.7 (− 2.37)	0.62 (0.51)
Vascular disorders (SOC: 10,047,065)	88	1.56 (1.27–1.93)*	1.55 (17.38)	0.63 (− 1.04)	1.55 (1.25)
Investigations (SOC: 10,022,891)	457	2.87 (2.6–3.17)*	2.59 (473.76)*	1.37 (− 0.29)	2.59 (2.34)*
Injury, poisoning and procedural complications (SOC: 10,022,117)	367	1.02 (0.91–1.14)	1.02 (0.13)	0.03 (− 1.64)	1.02 (0.91)
Blood and lymphatic system disorders (SOC: 10,005,329)	48	0.95 (0.71–1.26)	0.95 (0.15)	− 0.08 (− 1.75)	0.95 (0.71)
Cardiac disorders (SOC: 10,007,541)	29	0.47 (0.33–0.68)	0.48 (17.04)	− 1.07 (− 2.74)	0.48 (0.33)
Respiratory, thoracic and mediastinal disorders (SOC: 10,038,738)	117	0.85 (0.71–1.02)	0.85 (3.05)	− 0.23 (− 1.89)	0.85 (0.71)
Metabolism and nutrition disorders (SOC: 10,027,433)	29	0.49 (0.34–0.7)	0.49 (15.7)	− 1.03 (− 2.7)	0.49 (0.34)
Renal and urinary disorders (SOC: 10,038,359)	26	0.41 (0.28–0.6)	0.41 (21.93)	− 1.27 (− 2.94)	0.41 (0.28)
Infections and infestations (SOC: 10,021,881)	101	0.59 (0.49–0.72)	0.61 (27.55)	− 0.72 (− 2.39)	0.61 (0.5)
Psychiatric disorders (SOC: 10,037,175)	67	0.4 (0.31–0.51)	0.41 (59.06)	− 1.28 (− 2.94)	0.41 (0.32)
Musculoskeletal and connective tissue disorders (SOC: 10,028,395)	155	1.01 (0.86–1.18)	1.01 (0)	0.01 (− 1.66)	1.01 (0.86)
Eye disorders (SOC: 10,015,919)	48	0.83 (0.63–1.11)	0.84 (1.54)	− 0.26 (− 1.92)	0.84 (0.63)
Hepatobiliary disorders (SOC: 10,019,805)	42	1.69 (1.25–2.3)*	1.68 (11.76)	0.75 (− 0.92)	1.68 (1.24)
Ear and labyrinth disorders (SOC: 10,013,993)	15	1.19 (0.72–1.97)	1.19 (0.45)	0.25 (− 1.42)	1.19 (0.72)
Social circumstances (SOC: 10,041,244)	21	1.48 (0.96–2.28)	1.48 (3.27)	0.56 (− 1.1)	1.48 (0.96)
Reproductive system and breast disorders (SOC: 10,038,604)	13	0.65 (0.38–1.12)	0.65 (2.47)	− 0.62 (− 2.29)	0.65 (0.38)
Surgical and medical procedures (SOC: 10,042,613)	17	0.4 (0.25–0.65)	0.41 (15.04)	− 1.3 (− 2.97)	0.41 (0.25)
Product issues (SOC: 10,077,536)	15	0.28 (0.17–0.46)	0.28 (27.91)	− 1.83 (− 3.49)	0.28 (0.17)
Neoplasms benign, malignant and unspecified (incl cysts and polyps) (SOC: 10,029,104)	14	0.13 (0.08–0.22)	0.13 (82.51)	− 2.92 (− 4.58)	0.13 (0.08)
Immune system disorders (SOC: 10,021,428)	26	0.7 (0.47–1.03)	0.7 (3.4)	− 0.52 (− 2.18)	0.7 (0.48)
Pregnancy, puerperium and perinatal conditions (SOC: 10,036,585)	1	0.09 (0.01–0.63)	0.09 (9.44)	− 3.5 (− 5.17)	0.09 (0.01)
Congenital, familial and genetic disorders (SOC: 10,010,331)	2	0.23 (0.06–0.94)	0.23 (5.01)	− 2.09 (− 3.76)	0.23 (0.06)

*ROR* reporting odds ratio, *CI* confidence interval, *PRR* proportional reporting ratio, *χ*^*2*^ chi-squared, *IC* information component, *IC* 025 the lower limit of 95% CI of the IC, *EBGM* empirical Bayesian geometric mean, *EBGM* 05 the lower limit of 95% CI of EBGM.

*Indicates statistically significant potential signals in algorithm.

In total, disproportionality signals were identified for 44 PTs involved 17 SOCs conforming to the four algorithms simultaneously are shown in Table 3. In our statistical results, the most common AEs were platelet count decreased (20.2%, n = 165, PT: 10,035,528), headache (16.7%, n = 136, PT: 10,019,211), platelet count increased (11.9%, n = 97, PT: 10,051,608), platelet count abnormal (6.3%, n = 51, PT: 10,035,526), contusion (2.7%, n = 22, PT: 10,050,584), pulmonary embolism (2.3%, n = 19, PT: 10,037,377), and deep vein thrombosis (2.1%, n = 17, PT: 10,051,055). In this study, PTs that were reported at a high relative frequency were unlabeled in the avatrombopag product labeling^7^ were seasonal allergy (PT: 10,048,908), rhinorrhea (PT: 10,039,101), abnormal liver function (PT: 10,024,690), antiphospholipid syndrome (PT: 10,002,817), ear discomfort (PT: 10,052,137), and photopsia (PT: 10,034,962).

**Table 3:** Reporting potential signals for avatrombopag at the Preferred Term (PT) level in FAERS database.

SOC	Preferred terms (PTs)	Avatrombopag cases reporting PT	ROR (95% two-sided CI)	PRR (χ^2^)	IC (IC 025)	EBGM (EBGM 05)
Blood and lymphatic system disorders (SOC: 10,005,329)	Platelet disorder (PT: 10,035,532)	7	50.54 (24.02–106.33)	50.43 (337.25)	5.65 (3.98)	50.15 (26.92)
	Thrombocytosis (PT: 10,043,563)	8	44.26 (22.08–88.74)	44.15 (335.72)	5.46 (3.79)	43.94 (24.55)
	Antiphospholipid syndrome (PT: 10,002,817)	3	39.52 (12.71–122.9)	39.48 (112.02)	5.3 (3.63)	39.31 (15.21)
	Immune thrombocytopenia (PT: 10,083,842)	7	15.02 (7.15–31.55)	14.99 (91.23)	3.9 (2.24)	14.96 (8.04)
Vascular disorders (SOC: 10,047,065)	Embolism (PT: 10,061,169)	6	17.87 (8.02–39.85)	17.84 (95.2)	4.15 (2.49)	17.81 (9.1)
	Deep vein thrombosis (PT: 10,051,055)	17	8.41 (5.22–13.55)	8.37 (110.31)	3.06 (1.4)	8.36 (5.61)
	Thrombosis (PT: 10,043,607)	17	4.6 (2.86–7.42)	4.58 (47.66)	2.2 (0.53)	4.58 (3.07)
	Haemorrhage (PT: 10,055,798)	15	3.2 (1.93–5.32)	3.19 (22.59)	1.67 (0.01)	3.19 (2.09)
Gastrointestinal disorders (SOC: 10,017,947)	Gingival bleeding (PT: 10,018,276)	7	12.91 (6.15–27.12)	12.88 (76.62)	3.69 (2.02)	12.86 (6.91)
Renal and urinary disorders (SOC: 10,038,359)	Renal vein thrombosis (PT: 10,038,548)	4	145.91 (54.29–392.13)	145.72 (565.72)	7.16 (5.49)	143.4 (62.71)
Social circumstances (SOC: 10,041,244)	Patient dissatisfaction with treatment (PT: 10,076,571)	10	122.5 (65.57–228.86)	122.1 (1184.92)	6.91 (5.24)	120.47 (71.41)
General disorders and administration site conditions (SOC: 10,018,065)	Drug effect less than expected (PT: 10,083,365)	9	12.87 (6.68–24.76)	12.83 (98.06)	3.68 (2.01)	12.81 (7.41)
	Adverse drug reaction (PT: 10,061,623)	17	3.51 (2.18–5.66)	3.5 (30.36)	1.81 (0.14)	3.5 (2.35)
Skin and subcutaneous tissue disorders (SOC: 10,040,785)	Petechiae (PT: 10,034,754)	7	15.23 (7.25–31.99)	15.2 (92.7)	3.92 (2.26)	15.17 (8.15)
Immune system disorders (SOC: 10,021,428)	Graft versus host disease (PT: 10,018,651)	5	16.23 (6.75–39.06)	16.21 (71.22)	4.02 (2.35)	16.18 (7.76)
	Seasonal allergy (PT: 10,048,908)	4	4.85 (1.82–12.92)	4.84 (12.19)	2.27 (0.61)	4.84 (2.13)
Respiratory, thoracic and mediastinal disorders (SOC: 10,038,738)	Pulmonary embolism (PT: 10,037,377)	19	5.68 (3.62–8.92)	5.65 (72.79)	2.5 (0.83)	5.65 (3.87)
	Rhinorrhoea (PT: 10,039,101)	12	3.51 (1.99–6.18)	3.5 (21.41)	1.81 (0.14)	3.5 (2.18)
Surgical and medical procedures (SOC: 10,042,613)	Splenectomy (PT: 10,041,642)	3	78.69 (25.24–245.34)	78.61 (227.88)	6.28 (4.61)	77.94 (30.1)
Injury, poisoning and procedural complications (SOC: 10,022,117)	Incorrect dosage administered (PT: 10,073,768)	4	13.91 (5.21–37.1)	13.89 (47.77)	3.79 (2.13)	13.87 (6.1)
	Inappropriate schedule of product administration (PT: 10,081,572)	75	5.08 (4.04–6.39)	4.98 (239.66)	2.32 (0.65)	4.98 (4.11)
	Contusion (PT: 10,050,584)	22	5.04 (3.31–7.67)	5.01 (70.74)	2.33 (0.66)	5.01 (3.53)
	Prescribed overdose (PT: 10,051,076)	6	6.49 (2.91–14.46)	6.48 (27.77)	2.69 (1.03)	6.47 (3.31)
Nervous system disorders (SOC: 10,029,205)	Cerebral venous sinus thrombosis (PT: 10,083,037)	3	42.99 (13.82–133.73)	42.95 (122.33)	5.42 (3.75)	42.75 (16.54)
	Headache (PT: 10,019,211)	136	4.87 (4.1–5.79)	4.7 (399.44)	2.23 (0.56)	4.7 (4.07)
	Hepatic encephalopathy (PT: 10,019,660)	3	7.7 (2.48–23.9)	7.69 (17.45)	2.94 (1.27)	7.69 (2.98)
	Cerebral haemorrhage (PT: 10,008,111)	8	5.3 (2.65–10.6)	5.29 (27.79)	2.4 (0.73)	5.28 (2.96)
	Head discomfort (PT: 10,019,194)	5	5.27 (2.19–12.67)	5.26 (17.25)	2.39 (0.73)	5.26 (2.52)
Investigations (SOC: 10,022,891)	Platelet count increased (PT: 10,051,608)	97	149.51 (121.94–183.31)	144.74 (13,629.22)	7.15 (5.49)	142.45 (120.11)
	Platelet count abnormal (PT: 10,035,526)	51	156.46 (118.36–206.84)	153.84 (7614.21)	7.24 (5.57)	151.26 (119.75)
	Platelet count decreased (PT: 10,035,528)	165	32.83 (28.06–38.42)	31.09 (4797.52)	4.95 (3.29)	30.99 (27.17)
	Ammonia increased (PT: 10,001,946)	3	14.71 (4.74–45.66)	14.69 (38.22)	3.87 (2.21)	14.67 (5.68)
	Full blood count abnormal (PT: 10,017,412)	12	6.48 (3.68–11.43)	6.46 (55.35)	2.69 (1.02)	6.45 (4.02)
	Liver function test abnormal (PT: 10,024,690)	5	6.64 (2.76–15.97)	6.63 (23.9)	2.73 (1.06)	6.63 (3.18)
	General physical condition abnormal (PT: 10,058,911)	3	7.66 (2.47–23.79)	7.66 (17.35)	2.94 (1.27)	7.65 (2.97)
	Laboratory test abnormal (PT: 10,023,547)	8	4.9 (2.45–9.82)	4.89 (24.78)	2.29 (0.62)	4.89 (2.74)
Hepatobiliary disorders (SOC: 10,019,805)	Portal vein thrombosis (PT: 10,036,206)	10	78.18 (41.91–145.85)	77.93 (752.9)	6.27 (4.6)	77.27 (45.86)
	Autoimmune hepatitis (PT: 10,003,827)	3	8.95 (2.88–27.78)	8.94 (21.14)	3.16 (1.49)	8.93 (3.46)
Ear and labyrinth disorders (SOC: 10,013,993)	Ear discomfort (PT: 10,052,137)	4	8.48 (3.18–22.63)	8.47 (26.34)	3.08 (1.41)	8.47 (3.72)
Eye disorders (SOC: 10,015,919)	Photopsia (PT: 10,034,962)	3	12.34 (3.97–38.31)	12.33 (31.18)	3.62 (1.95)	12.31 (4.77)
Product issues (SOC: 10,077,536)	Product blister packaging issue (PT: 10,069,300)	3	50.49 (16.22–157.14)	50.44 (144.57)	5.65 (3.98)	50.16 (19.4)
	Product packaging difficult to open (PT: 10,079,403)	3	17.68 (5.69–54.91)	17.66 (47.07)	4.14 (2.47)	17.63 (6.83)
	Product packaging issue (PT: 10,069,405)	3	6.27 (2.02–19.47)	6.27 (13.27)	2.65 (0.98)	6.26 (2.43)
	Product supply issue (PT: 10,077,801)	3	5.64 (1.82–17.51)	5.64 (11.44)	2.49 (0.83)	5.63 (2.18)

*ROR* reporting odds ratio, CI confidence interval, *PRR* proportional reporting ratio, *χ*^*2*^ chi-squared, *IC* information component, *IC 025* *the* lower limit of 95% CI of the IC, EBGM empirical Bayesian geometric mean, *EBGM 05* the lower limit of 95% CI of EBGM.

### AE onset times

**Figure 2:**
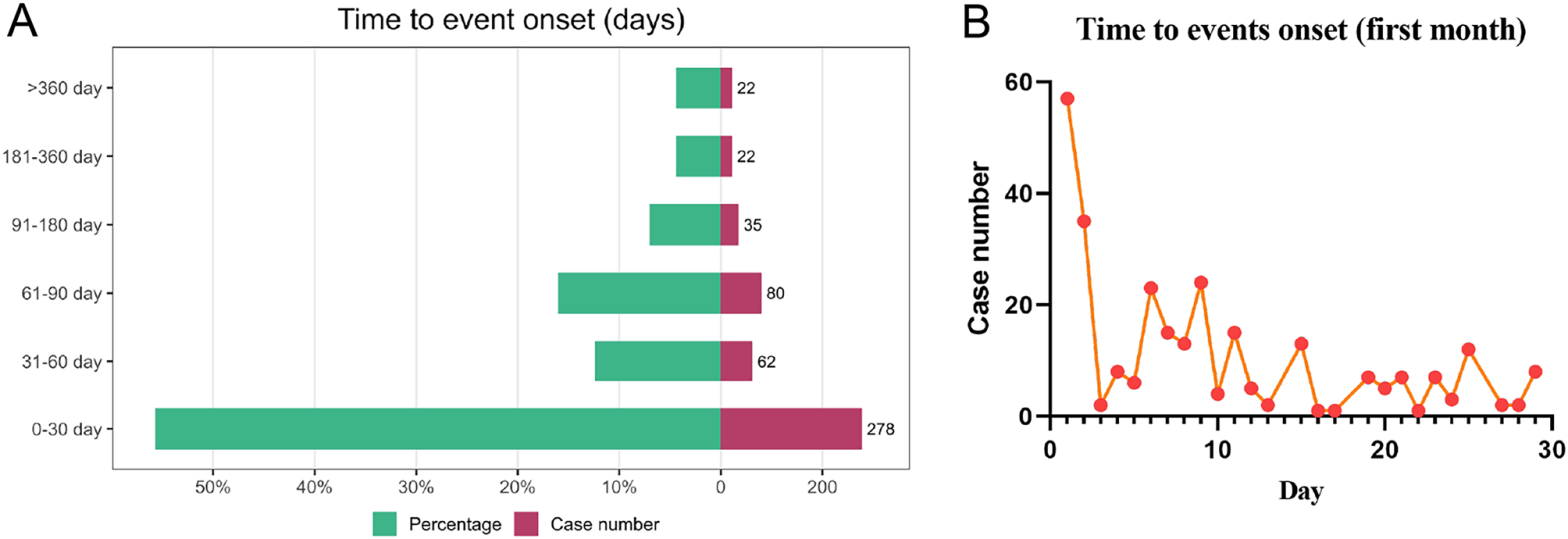
Time to onset of reported AEs. (**A**) Time to onset of reported AEs grouped by month. (**B**) Time to onset of reported AEs grouped by days with avatrombopag in the first month. *AE* adverse event.

The onset times of AEs reported with avatrombopag were extracted from the database. Patients whose time-to-onset analysis report fields in FAERS were blank or contained inaccurate information were excluded, 499 AEs’ onset times (41.2%) were reported (median 60 days). In approximately 55.7% of cases (n = 278), AEs occurred within the first month after initiation of avatrombopag (Fig. 2A). Additionally, the proportion of cases in which AEs occurred after 2 months (n = 62, 12.4%) and 3 months (n = 80, 16.0%) was significantly less than the number of AEs that occurred in the first month (*P* < 0.01), and the proportion of occurrences gradually decreased after 3 months. Furthermore, the highest number of AEs occurred on the first (n = 57, 20.5%) and second (n = 35, 12.6%) days after initiation of avatrombopag in the first month (Fig. 2B).

## Discussion

Avatrombopag absorption is not affected by dietary fat and divalent cations, it does not require subcutaneous injection, and it is an effective substitute for other TPO-RAs^10^. Research on avatrombopag has primarily focused on its mechanisms of action and clinical trials, with few studies having described the most recent real-world findings. Here we conducted a retrospective post-marketing pharmacovigilance study of avatrombopag using the FAERS data set.

AEs were more commonly reported in association with avatrombopag in women (54.3%) than in men (38.2%), probably because avatrombopag has been primarily approved for the treatment of otherwise poorly treated adult-onset primary chronic ITP, which is more prevalent in women than in men^17^. Our results show that AEs are more likely to be reported in association with avatrombopag among older adults, which is consistent with epidemiological data that older adults are more likely to develop ITP^18,19^. This has led to increased use of avatrombopag in women and older adults and, concomitantly, an increase in the probability of AEs. Clinical physicians should be aware of AEs associated with avatrombopag since its clinical use is increasing, especially among women and older patients. Our results reveal that AEs reported in association with avatrombopag that result in all serious outcomes that could lead to life-threatening conditions and death were 16.8%. Therefore, early identification of avatrombopag-associated AEs and avoidance of serious AEs are particularly important for the clinical use of avatrombopag.

In the FAERS database, disproportionality signals were identified for 44 PTs across 17 organ systems. The greatest relative frequency among all AEs reported in association with avatrombopag include headache, thrombosis, drug effects less than expected, abnormal laboratory test results, and others, which were consistent with the avatrombopag product labeling and previous results^20–22^.

There is a large number of AEs related to nervous system disorders that have been disproportionately reported in association with avatrombopag in FAERS. Our results indicate that headache was the most common AE, which is consistent with existing studies. Jurczak et al. found that headache was the most commonly reported AE (37.5% for the avatrombopag treatment group compared to 11.8% for the placebo group)^13^. However, a phase 3 clinical trial on the use of avatrombopag for treating ITP in China showed that headache was the third most common AE (7/48, 14.6%)^23^; the reason for this may be ethnic differences or a small sample size, and the relationship still needs to be further explored.

For blood and lymphatic system disorders, thrombocytosis is one of the most prominent reported AEs in the present study. A recent study that focused on the difference in the role of avatrombopag between acute and chronic ITP reported that thrombocytosis occurred in 44% of patients (10/23) in the newly diagnosed/persistent ITP group and 40% of patients (21/53) in the chronic ITP group, with no significant difference between the two groups^24^. Interestingly, antiphospholipid syndrome has been revealed as an AE of avatrombopag in the previously published literature. Van de Vondel et al. reported that a 20-year-old patient with chronic ITP developed antiphospholipid syndrome 3 weeks after treatment with avatrombopag^25^. Patients with antiphospholipid syndrome are at a high risk of venous thrombosis^26^; therefore, serological tests for antiphospholipid syndrome should be performed for chronic patients with ITP.

Thrombosis—a common etiology in myocardial infarction, ischemic stroke, and venous thromboembolism—is responsible for 1 in 4 deaths worldwide^27^. We found statistically through the FARS database that thrombosis reported in association with avatrombopag in multiple organ systems, including renal vein thrombosis (n = 4) in renal and urinary disorders; pulmonary embolism (n = 19) in respiratory, thoracic, and mediastinal disorders; portal vein thrombosis (n = 10) in hepatobiliary disorders; and deep vein thrombosis (n = 17) in vascular disorders. In a randomized trial of avatrombopag, five venous thrombotic events were reported in four patients: iliac deep vein thrombosis, stroke, superficial thrombophlebitis, myocardial infarction, and retinal artery occlusion^28^. However, patients taking avatrombopag were not at an increased risk of thrombotic events in the ADAPT-1 and ADAPT-2 trials, but these trials were not powered to assess thrombotic risk^29^. Of all AE events, thrombosis requires extra attention.

Unexpected and significant potential signals were detected in our analysis, including seasonal allergy, rhinorrhea, abnormal liver function test outcomes, antiphospholipid syndrome, ear discomfort, and photopsia. Abnormal liver function test outcomes seem to result from the patients’ primary disease rather than from drug-induced AEs^7^. A review of the literature did not reveal any new significant signaling-related reports. Therefore, more clinical studies are needed to establish the pathogenesis of these AEs.

We report a median time to the onset of AEs of 60 days after avatrombopag initiation, with most cases occurring within the first month (n = 278, 55.7%). In the first month after treatment, AEs were reported on the first (n = 57, 20.5%) and second days (n = 35, 12.6%), and then they gradually stabilized. These results suggest that special attention must be paid to avatrombopag-associated AEs in the first month of treatment, especially on the first and second days after taking the drug, to ensure the safety of patients to the greatest extent.

Despite an increase in the number of some reported AEs was associated with the use of avatrombopag in the FAERS database, our study has some limitations. Firstly, the FAERS database is a worldwide spontaneous reporting system. It has some inherent selection biases, such as the fact that reported cases are not fully documented. AEs can also be reported by consumers since voluntary reporting is not limited to health care professionals; this may lead to a lack of professionalism in some reported AEs. Secondly, there may be controversy over some of the relevant AEs since the FDA does not require proof of causation. Similarly, we cannot prove causality between avatrombopag and the reported AEs. Thirdly, the lack of information on healthy populations makes it impossible to calculate the incidence of drug-related AEs. Fourthly, owing to the lack of information in the FAERS database, confounding factors such as age, comorbidities, or other factors were not controlled for in this study. The disproportionality algorithms used provide crude measures and no adjusted analyses were performed. Fifthly, due to limitations of the FAERS database, this study did not carry out correlation analyses when data were collected from the same locations or countries, which may have led to biased results. Data mining is not a substitute for expert review; however, its advantages can become apparent when dealing with large amounts of data, which are analyzed to make the results more comprehensive^15^. The FAERS database in pharmacovigilance studies has substantial limitations, but an analysis of AEs reported in association with avatrombopag revealed some unexpected potential AE signals that can inform future clinical studies. Avatrombopag must still be monitored on an ongoing basis for its efficacy and safety.

## Conclusion

We used the FAERS database to systematically analyze AEs reported in association with avatrombopag and their onset time after administration. Unexpected AEs such as seasonal allergy, rhinorrhea, antiphospholipid syndrome, ear discomfort, and photopsia could be reported. Common reported AEs include headache, contusion, thrombocytosis, and thrombosis, which should be taken seriously. The results of this study potentially provide valuable information for clinical monitoring and identifying risks associated with avatrombopag.

### Supplementary Information

Supplementary Table S1.

## Data Availability

The data sets generated during the current study are available from the corresponding author on reasonable request.

## Abbreviations

AEs: Adverse events
FAERS: Food and drug association adverse event reporting system
FDA: Food and drug administration
ITP: Immune thrombocytopenia
PTs: Preferred terms
SOC: Social organ class
TPO: Thrombopoietin
TPO-RA: Thrombopoietin receptor agonist
