# Supplementary material for: Data mining and safety analysis of avatrombopag: a retrospective pharmacovigilance study based on the US food and drug administration’s adverse event reporting system
Source: Sci Rep. 2024 May 17;14:11262. doi: 10.1038/s41598-024-62129-5 (PMC11101459; doi:10.1038/s41598-024-62129-5)
Supplement: Supplementary file 1 — Supplementary Table S1. [file 41598_2024_62129_MOESM1_ESM.docx]

**Supplementary Table 1. Four major algorithms used for signal detection.**

| Algorithms | Equation | Criteria |
| --- | --- | --- |
| ROR | ROR=(a/c)/(b/d)=ad/bc | lower limit of 95% CI>1, N≥3 |
|  | 95%CI=e^ln(ROR)±1.96(1/a+1/b+1/c+1/d)^0.5^ |  |
| PRR | PRR=[a/(a+b)]/[c/(c+d)] | PRR≥2, χ^2^≥4, N≥3 |
|  | χ^2^=[(ad-bc)^2](a+b+c+d)/[(a+b)(c+d)(a+c)(b+d)] |  |
| BCPNN | IC=log_2_(a(a+b+c+d)/(a+b)/(a+c)) | IC_025_>0 |
|  | IC_025_=e^ln(IC)−1.96(1/a+1/b+1/c+1/d)^0.5^ |  |
| MGPS | EBGM=a(a+b+c+d)/ [(a+c)(a+b)] | EBGM05>2, N>0 |
|  | 95%CI=e^ln(EBGM)±1.96(1/a+1/b+1/c+1/d)^0.5^ |  |

Equation: a, number of reports containing both the target drug and target adverse drug reaction; b, number of reports containing other adverse drug reaction of the target drug; c, number of reports containing the target adverse drug reaction of other drugs; d, number of reports containing other drugs and other adverse drug reactions. 95%CI, 95% confidence interval; *N*, the number of reports; χ^2^, chi-squared; IC, information component; IC_025_, the lower limit of 95% CI of the IC; EBGM, empirical Bayesian geometric mean; EBGM05, the lower limit of 95% CI of EBGM.
